# Enhanced oxygen evolution reaction on amine functionalized graphene oxide in alkaline medium†

**DOI:** 10.1039/c8ra10286d

**Published:** 2019-02-22

**Authors:** Vijay S. Sapner, Balaji B. Mulik, Renuka V. Digraskar, Shankar S. Narwade, Bhaskar R. Sathe

**Affiliations:** Department of Chemistry, Dr. Babasaheb Ambedkar Marathwada University Aurangabad 431004 Maharashtra India bhaskarsathe@gmail.com

## Abstract

Development of highly efficient oxygen evolution reaction (OER) electrocatalysts is a critical challenge in the cost-effective generation of clean fuels. Here, a metal-free tyramine functionalized graphene oxide (T-GO) electrocatalyst is proposed to use in alkaline electrolytes for enhanced OER. Moreover, the T-GO and GO nanomaterials are well characterized by SEM, XRD, FTIR, XPS and Raman spectroscopy. T-GO exhibits an electrocatalytic OER with a current density of 2 mA cm^−2^ at a low onset potential of ∼1.39 V and a small Tafel slope of about 69 mV dec^−1^ and GO exhibits an onset potential of 1.51 V and Tafel slope of about 92 mV dec^−1^. Additionally, the current stability and RRDE based diffusion controlled response of the T-GO electrocatalyst are outstanding compared to GO. This study establishes metal free T-GO as an efficient electrocatalyst for the OER and used for cathodic production of hydrogen as a counter reaction in the field of water splitting.

## Abstract 2

Metal free tyramine functionalized graphene oxide (T-GO) is a promising electrocatalyst for oxygen evolution reaction (OER) in alkaline medium having high activity and stability, resulting from the tyramine active sites.

## Graphical abstract

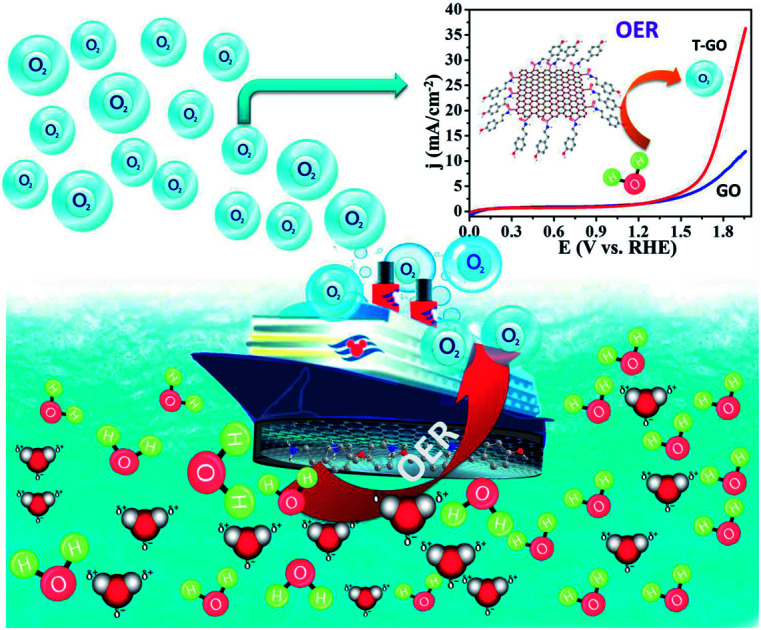

## 1. Introduction

Energy crises and environmental pollution are among the most important issues worldwide and affect every country. Numerous efforts have been devoted to an alternative path for renewable energy, such as solar cells, chemical to electrochemical energy conversion *etc.*^1^ In the electrochemical hydrogen evolution reaction (HER), H_2_ is produced as an efficient energy carrier for possible future energy systems.^2^ Unfortunately, however, the electrochemical oxygen evolution reaction (OER) is a great challenge in the development of power sources because of its complex mechanism of multi-electron transfer steps and the poor stability of the electrode materials.^3^ The overall water splitting reaction involves the production of H_2_ and O_2_ through HER at the anode and OER at the cathode respectively.^4^ Electrocatalytic water splitting (electrolysis) is currently an attractive alternative strategy for energy generation using water and electricity at room temperature.^5^ Water splitting is kinetically hindered by the electron transfer rate due to the unequal electron transfers at the anodic and cathodic compartment, *i.e.* OER corresponds to four electron transfer (4OH^−^ → O_2_ + 2H_2_O + 4e^−^) while HER is a two electron transfer reaction (2H^+^ + 2e^−^ → H_2_).^6,7^ In particular, a high input of kinetic energy to the OH^−^ at the anode and a catalyst with highly abundant active sites are usually required to reduce the overpotential for the OER.^8^

In recent years, the synthesis of a variety of electrocatalytic systems for efficient OER has been reported in the literature, including transition metals,^9^ bimetallic nanomaterials,^10^ metal oxide nanocomposites,^6,11^ polymer grafted nanomaterials,^12,13^ carbon-based nanocomposites,^14–20^ heteroatom doped nanomaterials, noble metal free nanocomposites^21–26^ and many more. In particular, Ir and Ru based metal nanoparticles and composites have been widely investigated and achieved some of the highest electrocatalytic activities towards OER. However, their use on large scales as an electrode material is seriously hindered by their high cost, which has prevented their commercialization. Therefore, recent research has tended to focus on development of alternatives to these expensive metal based systems, for example, carbon nanostructures with heteroatoms like sulphide, phosphide, metal oxides, *etc.* These materials are cost effective and have been commercialized.^10*b*,18,26^ Nanostructured catalysts are of great utility because of the reduced electrode content and high catalytically active surface area achieved by a large surface area to volume ratio. Accordingly, it is an extremely important challenge to develop new, facile and highly reproducible electrocatalytic systems for further enhancement of the OER performance using metal-free carbon-based nanomaterials with abundant active sites and large surface area at the nanoscale. These strategies are also important to further improve the performance of metal-free electrocatalysts for large scale overall water splitting, energy conversion and fuel cell applications.^13,22,23,27,28^

Herein, we demonstrate the highly efficient metal-free tyramine functionalized graphene oxide (T-GO) electrocatalyst with enhanced electrocatalytic activity towards OER. The active functionality of tyramine facilitates the electron transfer reaction at the graphene interface^29^ and increases the performance towards OER. Our electrochemical findings suggest that the metal-free tyramine functionalized T-GO electrocatalyst has higher OER activity in an alkaline medium as compared to GO. We propose a plausible electrochemical mechanism to explain the better performance of the T-GO electrocatalyst for OER. More significantly, our electrochemical OER catalyst in alkaline medium outperforms other reported systems from the literature.

## 2. Experimental section

### 2.1 Chemicals

Graphite fine powder (extra pure), sulphuric acid (H_2_SO_4_), nitric acid (HNO_3_), thionyl chloride (SOCl_2_), tetrahydrofuran (THF), dimethylformamide (DMF), hydrochloric acid (HCl), potassium hydroxide (KOH) and acetone, all of analytical grade, were purchased and used without any further purification. Tyramine (99%) was purchased from Sigma-Aldrich. Deionized (DI) water (18 MΩ) from a Milli-Q system was used for all syntheses, purification of electrocatalysts and electrochemical studies.

### 2.2 Material synthesis

Graphene oxide (GO) was synthesized from graphite powder by the modified Hummers method.^30^ The typical synthesis procedure of GO and T-GO was reported in previous work.^29^ In more detail, 1 g of graphite powder was dispersed in 3 : 1 H_2_SO_4_ : HNO_3_ in a round bottom flask, and the mixture was stirred in an ice bath for 30 min and sonicated further for 6 h at room temperature. After this time, a black-yellow aqueous suspension appeared. This suspension was further refluxed for 24 h followed by natural cooling and was then filtered and washed with 1 M HCl followed by DI water and dried in an oven for 30 min.

**Scheme 1:**
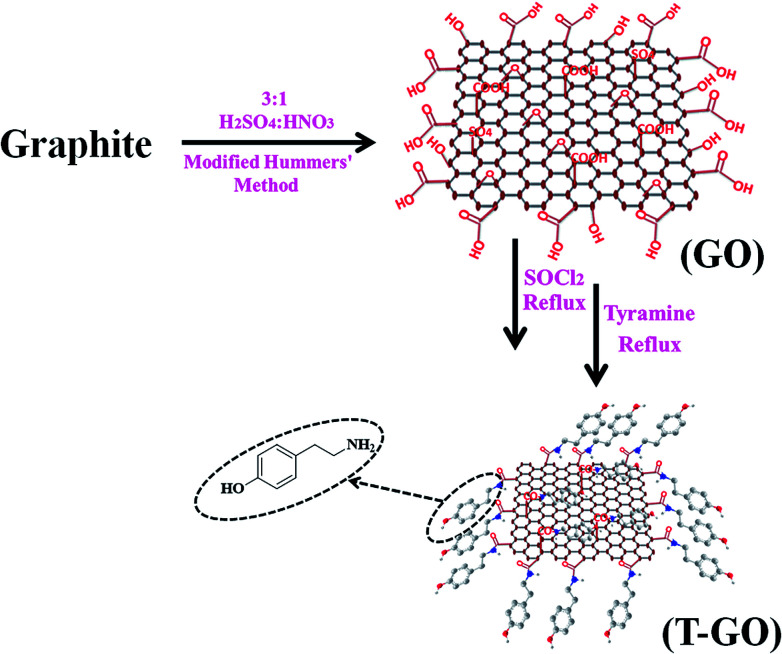
Schematic representation of tyramine functionalized graphene oxide (T-GO).

200 mg of the above as-synthesized GO was taken, then 20 mL of SOCl_2_ followed by 1 mL of DMF were added under cool conditions, and this mixture was stirred for 1 h followed by reflux for 24 h. For further functionalization with tyramine, 20 mg of the above GO-COCl was taken and added into a solution of 10 mg of tyramine in 20 mL of DMF and sonicated for 30 min and then refluxed for 12 h. This T-GO suspension was filtered and washed to remove unbound tyramine and other water soluble impurities by using DI water followed by alcohol. A schematic representation of the assembling procedure for T-GO is shown in Scheme 1.

### 2.3 Material characterization

X-ray diffraction (XRD) was carried out on a Rigaku Ultima IV fully automatic high-resolution X-ray diffractometer system, with an X-ray generator operating at 40 kV and 40 mA at a 2*θ* step of 0.01° at room temperature. FTIR spectra were measured in the 4000–400 cm^−1^ range on a PerkinElmer Spectrum-I spectrometer with samples prepared as KBr pellets. Scanning electron microscopic (SEM) measurements were carried out with a JEOL (JSM-7600F) instrument equipped with an energy dispersive analysis of X-ray (EDAX) attachment. Raman spectroscopy was performed by a Raman spectrometer with microscope (Seki Technotron Corporation, Tokyo) with 532 nm laser equipped with X-ray photoelectron spectroscopy (XPS).

### 2.4 Electrocatalytic measurement

The electrocatalytic performance of the GO and T-GO catalysts was evaluated by using a rotating ring-disk electrode (RRDE) to perform cyclic voltammograms (CVs), linear sweep voltammetry (LSV), and chronoamperometry on a CHI-660E (CH-Instruments, USA) using a conventional three-electrode system. Preliminarily, the glassy carbon (GC) surface (3 mm in diameter) was cleaned by polishing with three different sizes (1.0, 0.3, 0.05 μm) of alumina powders simultaneously. Then, this polished GC electrode was sonicated in DI water followed by methanol to remove residual alumina particles and other organic and inorganic impurities. The T-GO modified GC electrode was used as the working electrode, with Pt wire and saturated calomel electrode (SCE) as the counter and reference electrodes respectively.

The GC electrode was modified using 2 mg of T-GO dispersed in 0.1 mL of isopropyl alcohol. The electrode was sonicated for 1 h and a roughly 6 μL drop of T-GO suspension was drop-casted on the cleaned GC electrode and dried at room temperature. The experiments were carried out at room temperature in 0.5 M KOH solution. All potentials were measured *vs.* the SCE and normalized to the reversible hydrogen electrode (RHE).

## 3. Results and discussion

**Fig. 1:**
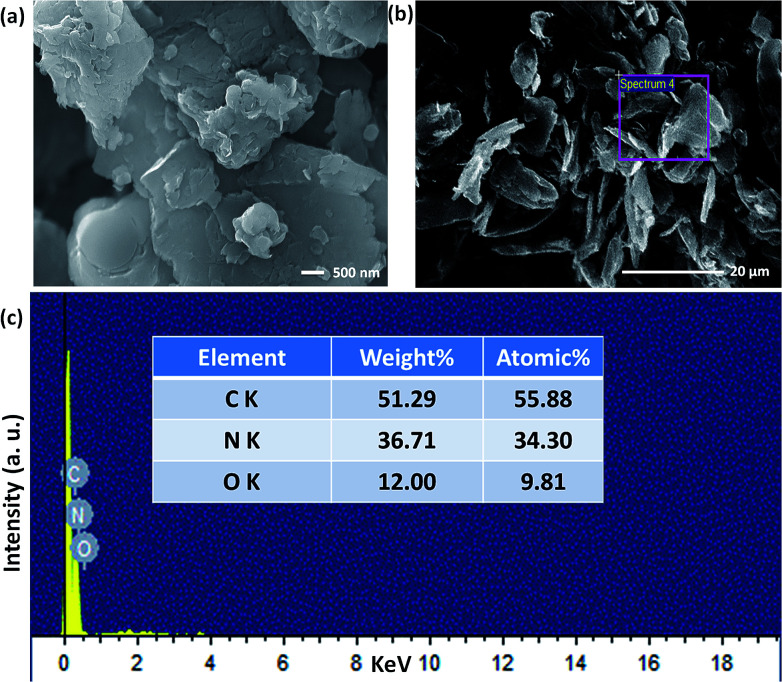
Scanning electron microscopic images of (a) and (b) T-GO (different magnification) and (c) EDAX of T-GO confirms C, N and O.

The structural and morphological properties of as-synthesised GO and T-GO were investigated by different microscopic and spectroscopic characterization techniques. Accordingly, Fig. 1(a) and S1† represent SEM images of T-GO and GO, both having ∼20 nm width and average sheet diameter of more than 100 nm. Although in both cases the sheets have similar dimensions, the smaller separation between sheets found in T-GO could be due to the additional functionality of tyramine on graphene, and is in good agreement with the literature on similar systems.^26,27^

Fig. S7† shows the typical sheet like morphology of GO as imaged by transmission electron microscopy (TEM).

**Fig. 2:**
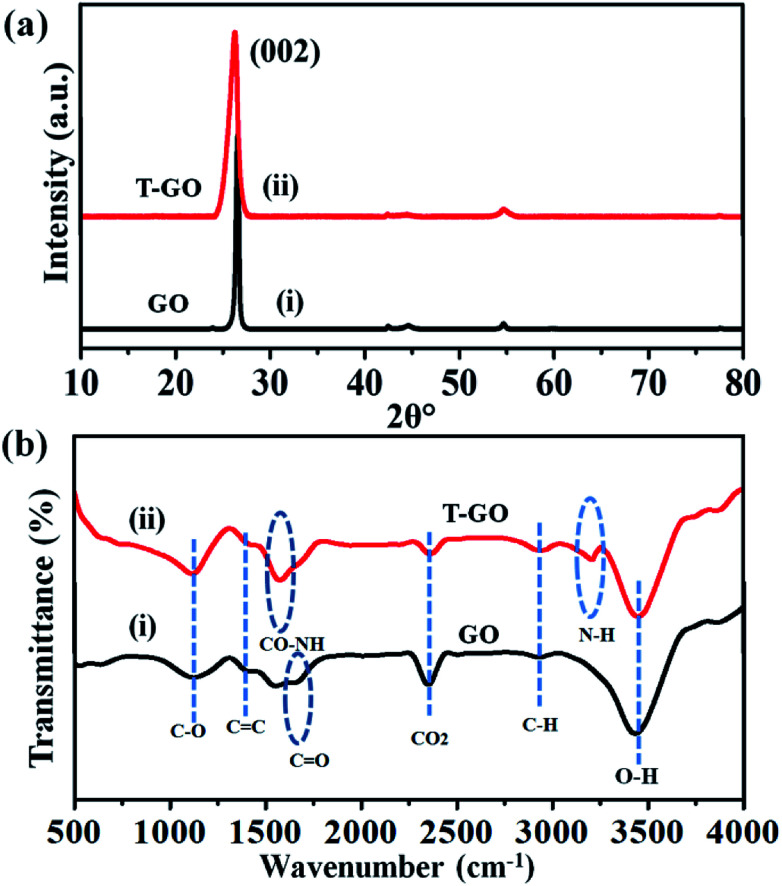
Superimposed (a) XRD patterns and (b) FTIR spectra of as-prepared GO and T-GO.

Moreover, the representative SEM image of T-GO shown in Fig. 1(b) also supports the above conclusion. To further confirm the chemical composition and information about tyramine functionalization on GO, EDAX analysis was performed, as displayed in Fig. 1(c). Accordingly, the strong signals in the C, O, and N regions confirm the surface functionalization of tyramine on GO. Further, the representative superimposed XRD patterns of GO and T-GO shown in Fig. 2(a) contain the standard diffraction peaks for the (002), (101) and (110) planes of GO, showing that in T-GO, negligible changes occurred after functionalization.^29,31^ The FTIR spectra of GO and T-GO are shown in Fig. 2(b), where the sharp peaks corresponding to O–H stretching centred at 3442 cm^−1^ are related to adsorbed water molecules and also associated with alcoholic and carboxylic acid functional groups. Moreover, the presence of other functional groups on the surface of GO can also be confirmed, for example, the C=C, C=O, C–O, and C–H bond stretching bands at 1639 cm^−1^, 1710 cm^−1^, 1107–1396 cm^−1^, and 2917 cm^−1^ respectively correspond to carbon skeleton and oxidative functionalities.^32,33^ After surface functionalization of GO with tyramine, the intensities of hydroxy groups and the C=O stretching band decrease, and representative stretching bands are observed at 1580 cm^−1^ and 3210 cm^−1^, corresponding to CO–NH and N–H of T-GO.^34–36^

**Fig. 3:**
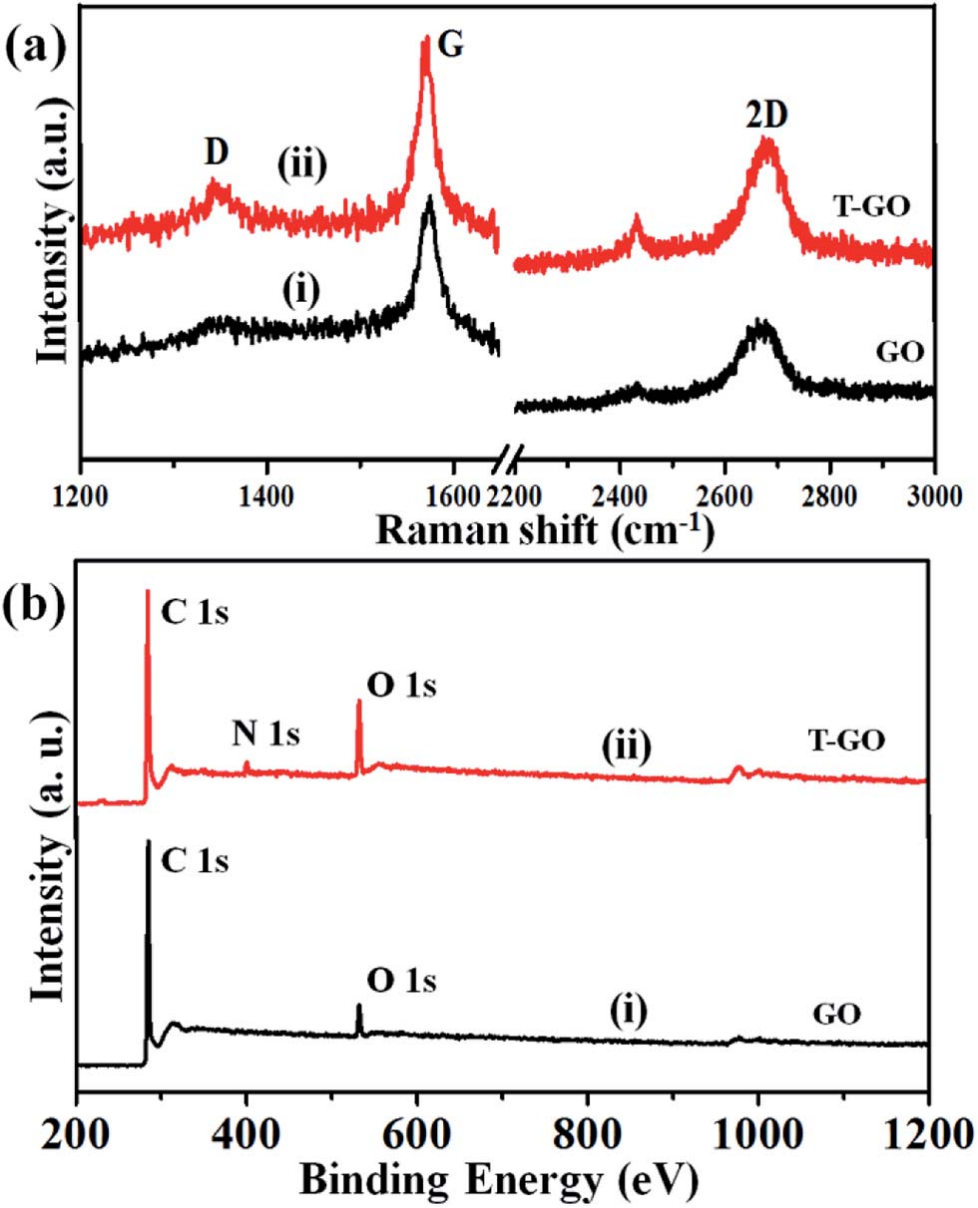
(a) Raman spectra and (b) full scan XPS spectra for GO (i) and T-GO (ii).

Raman spectroscopy is the most immediate and non-destructive method to describe the structure and nature of carbon-based materials. Accordingly, Fig. 3(a) represents the superimposed Raman spectra for GO and T-GO, with representative bands at 1345 cm^−1^ and 1343 cm^−1^ for the D band and 1572 cm^−1^ and 1570 cm^−1^ for the G band for GO and T-GO respectively.^37–39^ Also, the characteristic 2D bands of T-GO and GO are at 2677 cm^−1^ and 2669 cm^−1^ respectively. Interestingly, the intensity ratio *I*_D_/*I*_G_ for GO and T-GO is 0.18 and 0.32 respectively.^40,41^ This increase in *I*_D_/*I*_G_ ratio for T-GO suggests a greater degree of disorder in T-GO after functionalization.^42^

The GO and T-GO were further characterized by XPS. Accordingly, high-resolution C 1s spectra for GO and T-GO are shown in Fig. S2(a),† where the spectral signals obtained at 284.8 eV, 286.1 eV, and 289.1 eV correspond to the C–C, C–O, and (C(O)OH) groups respectively for the GO.^43–45^ Moreover, after the functionalization by tyramine, an additional signal is observed at 286.1 eV, corresponding to C–N, as shown in Fig. S2(b)† for the T-GO. This confirms that tyramine functionalization occurs through amide (CO–NH) linkages and is also in good agreement with the above FT-IR findings. Moreover, the O 1s spectra of GO and T-GO, featuring peaks at 532.9 eV, 531.3 eV, 532.2 eV and 533.6 eV corresponding to the C–OH, C–O–C, C=O, and C–OH groups respectively, are shown in Fig. S3(a) and (b).†^29,46,47^ Meanwhile, the N 1s spectrum for T-GO shown in Fig. S4† was obtained to probe the surface functionalization of tyramine on the graphene oxide surface of T-GO. Accordingly, the representative signals at 399.5 eV and 401.0 eV correspond to the NH–C=O and C–N groups respectively.^48–50^ The wide scan XPS spectra of GO (i) and T-GO (ii) shown in Fig. 3(b) confirm the functionalization of tyramine on GO from the additional signal corresponding to N appearing in T-GO.

Further, thermogravimetric analysis (TGA) for GO and T-GO was performed under nitrogen atmosphere in the range of 30 to 1000 °C with temperature ramp rate of 20 °C min^−1^ (Fig. S5†). For GO and T-GO there are three stages of weight loss. In GO the weight loss in the first stage at about 387.71 °C is the removal of epoxy and oxygen containing functional groups associated with water molecules. The weight loss in the second stage at 591.30 °C involves 80% of the remaining mass, including functionalities like sp^3^ hybridised carbon atoms and phenolic groups that are also present on the surface of GO. After the functionalization by tyramine on the surface of GO, the thermal stability increases and the main weight loss occurs at 466 °C. This could be due to additional stability from amide formation and is in good agreement with similar studies.^50^ These results indicated the high degree of oxidation of the chemically prepared graphite in GO and T-GO. Thus, we can infer that the oxygen moieties are replaced by tyramine molecules after the amidation reaction of GO, in agreement with the above findings, especially Raman data.

AFM imaging was used to characterize the surface morphology and thickness of GO. Fig. S8† shows the AFM three dimensional image of GO, clearly showing sheets with some wrinkles and an average thickness of about 0.30 nm, indicating formation of single layered GO nanosheets.^51^

### 3.1 Electrochemical studies and reaction mechanism of OER

**Fig. 4:**
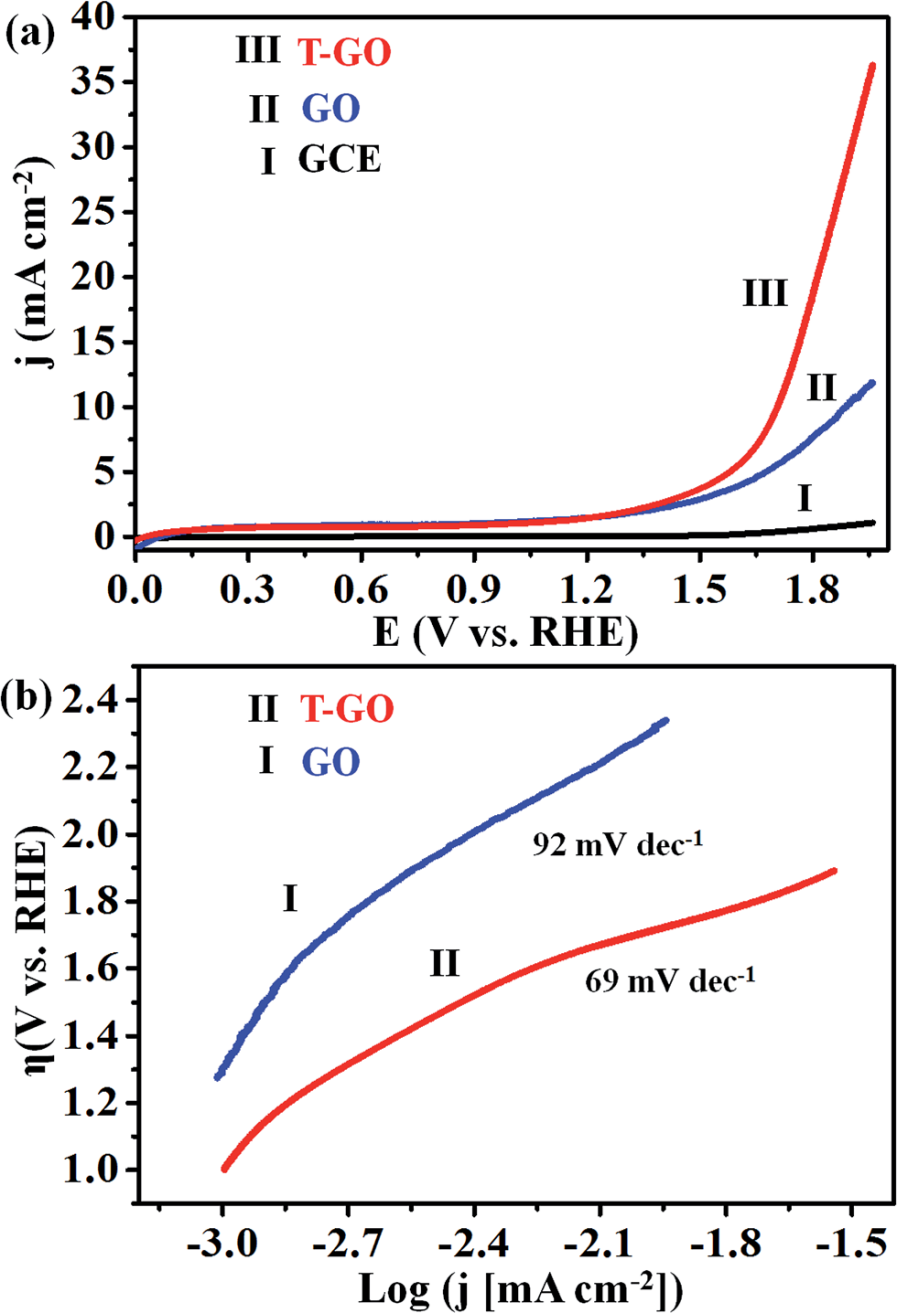
Superimposed (a) LSV of (I) GCE (bare), (II) GO and (III) T-GO, (b) Tafel plots for (II) T-GO and (I) GO in 0.5 M KOH using SCE and Pt foil as reference and counter electrodes respectively (all potentials were normalized with RHE) at 50 mV s^−1^.

Electrocatalytic studies of GO and T-GO based electrocatalytic systems were performed in solutions of 0.5 M KOH as shown in Fig. 4(a).

Accordingly, the LSV measurements show that T-GO shows excellent electrocatalytic activity compared with GO/GCE and also several times better than bare GCE. Interestingly, due to the tyramine functionalization on GO, T-GO exhibits a higher density of active sites and larger surface area than GO. For example, the LSV polarization curve for T-GO affords a current density of 2 mA cm^−2^ at an ultralow onset potential of ∼1.39 V *vs.* RHE, which is lower than that of GO and GCE. After the functionalization of tyramine on GO, the current density is significantly increased and the onset potential is shifted to a lower value for the OER. The corresponding Tafel plot of T-GO is shown in Fig. 4(b), and the Tafel slopes for GO and T-GO are found to be 92 and 69 mV dec^−1^ respectively, signifying that the T-GO has a considerable electrocatalytic activity towards OER as a metal-free electrocatalyst. The lower Tafel slope for the T-GO shows more facile charge transfer after functionalization of tyramine at the GO interface. This is in good agreement with earlier studies, and suggests a better performance than the reported metal free systems in the literature. Accordingly, Table 1 shows the comparative data of T-GO with other electrocatalysts using parameters like overpotential and their Tafel slope values. As displayed, the overpotential of the T-GO metal-free electrocatalyst is 176 mV at 2 mA cm^−2^ and the Tafel slope is 69 mV dec^−1^. In comparison with the metal-free electrocatalysts and metal-supported nanocomposites in the literature, T-GO shows excellent electrochemical OER performance. Moreover, the CVs for GO and T-GO were also recorded, as shown in Fig. S6,† and the comparative data are in line with the above findings.

**Table 1:** Comparative study of T-GO with other similar electrocatalysts towards oxygen evolution reaction (OER) in aq. KOH as an electrolytic solution

Carbon based catalyst system (nm)	Electrolyte concentration	Overpotential (mV *vs.* RHE)	Tafel slope (mV dec^−1^)	Ref.
NFPGNS	1 M	NA	78	4*c*
NGSHs	0.1 M	NA	83	20
CNTBN5-750	0.1 M	NA	122	22*a*
CuNi–C	1 M	408	83	54
Co/Mo	0.1 M	270	87	55
NiSe_2_/Ti	1 M	295	82	56*a*
Co_5_MnLDH/MWCNT	1 M	300	73.6	56*a*
PEG-CoO	0.1 M	348	79	57
NiMoO-SP/Ti	1 M	280	85	58
Co–Ni–B@NF	NA	313	120	59
T-GO	0.5 M	176	69	This work

**Fig. 5:**
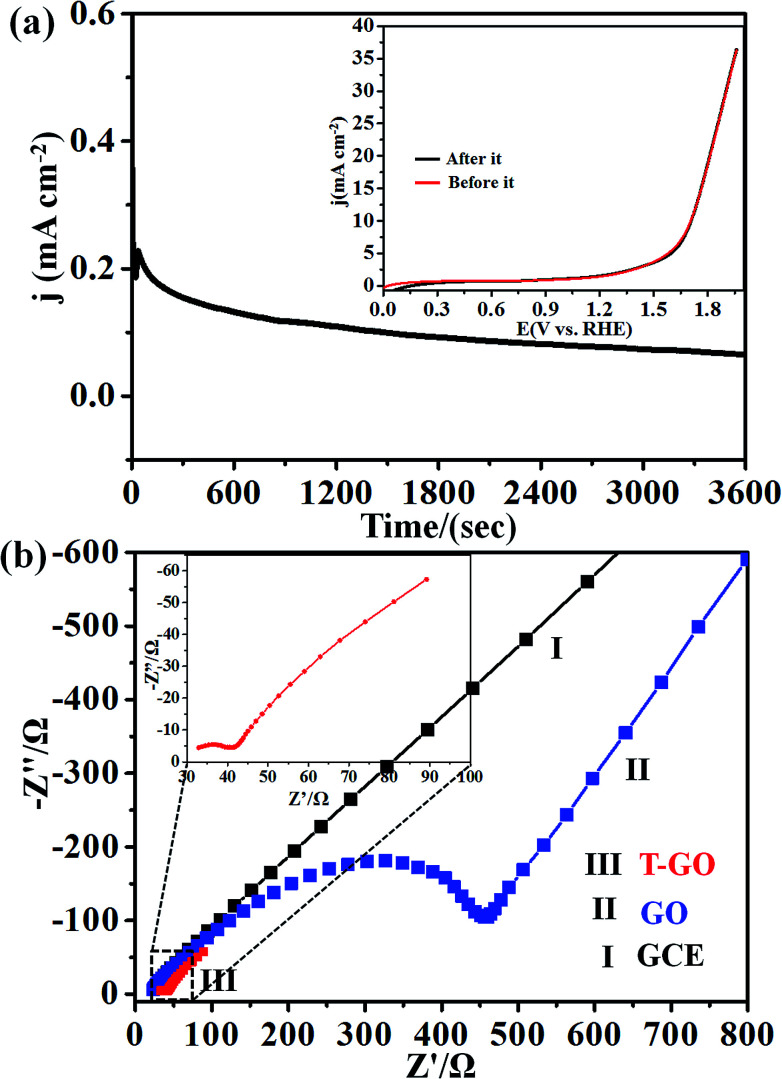
(a) Electrochemical chronoamperometric current stability at applied potential 1.39 V *vs.* RHE for 3600 s; inset shows superimposed LSV curves before and after these stability measurements for T-GO at 50 mV s^−1^. (b) Superimposed Nyquist plots for (I) GCE, (II) GO and (III) T-GO in 0.5 M KOH solution at applied potential of 1.39 V *vs.* RHE.

Next, the stability of T-GO was investigated further by chronoamperometric measurement at an applied potential of 1.39 V *vs.* RHE for 3600 s as shown in Fig. 5(a). Moreover, the inset of Fig. 5(a) shows the superimposed LSV curves before and after chronoamperometric measurement, which demonstrates there is no significant change in onset potential and current density after and before electrolysis for 3600 s. These findings suggest the excellent stability and reproducibility of the T-GO metal-free electrocatalyst during the OER, in good agreement with similar reports in the literature on other OER electrocatalysts. The comparison of metal-free T-GO and other electrocatalysts is summarized in Table 1 and clearly shows that compared with those electrocatalysts in the reported literature, TGO has excellent performance for OER.^51–59^ This promoted activity could be due to the availability of additional surface-active sites due to tyramine, which supports enhanced electron transfer capabilities.^29^

**Scheme 2:**
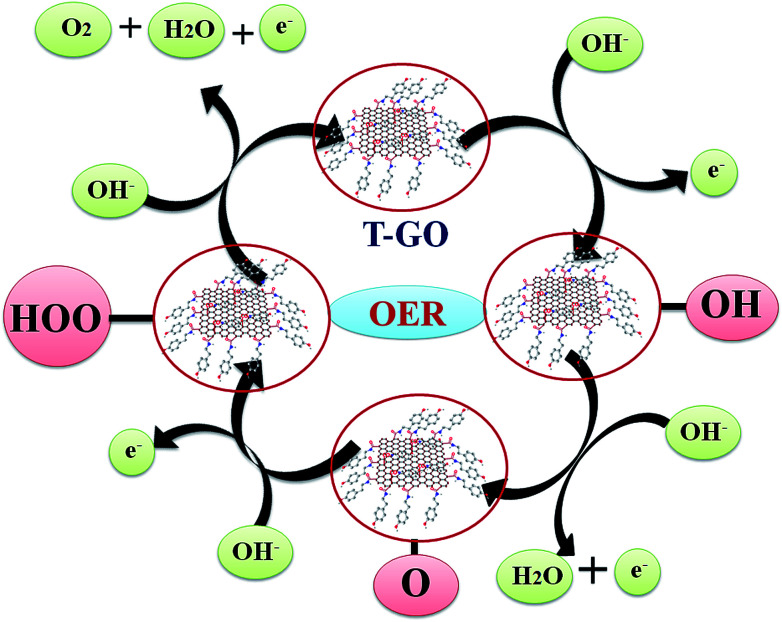
Reaction mechanism for probable electron transfer pathways for OER on T-GO in 0.5 M KOH.

The probable mechanistic pathway for OER on the surface of T-GO is shown in Scheme 2. Interestingly, herein the active sites of T-GO for electrocatalytic OER involve four consecutive one electron oxidation processes. In the first step of the reaction, a hydroxyl (OH) radical is adsorbed on the active site of T-GO, which results in formation of the intermediate T-GO-OH, by oxidation (removal of 1e^−^) of a hydroxide anion. This is followed by the formation of T-GO-O^−^ by the removal of a proton and an electron simultaneously from the T-GO-OH species. In the next step, a hydroxyl anion combines with T-GO-O^−^ to give the hydroperoxide intermediate T-GO-OOH. Then, proton-coupled electron transfer results in the O_2_ molecule species.^6*b*,9*b*,10*c*,27*a*,60–62^ The electrochemical mechanisms of OER in metal free systems are more complex than in metal systems.

In addition, the OER kinetics of the GO and T-GO electrode/electrocatalyst interface was studied *via* electrochemical impedance spectroscopy (EIS). Fig. 5(b) shows the Nyquist plots for bare GCE, GO and T-GO, each featuring two semicircles. The semicircles at the low- and high-frequency range in the Nyquist plots are attributed to the internal resistance *R*_s_ and charge transfer resistance *R*_ct_ respectively. The semicircle at low frequency is significantly reduced in surface functionalized electrocatalysts.^63–65^ Accordingly, T-GO showed a lower *R*_ct_ value than GO, suggesting a lower frequency and higher charge transport efficiency for T-GO as compared to GO. This implies that the tyramine functionality enhances the electrocatalytic activity by boosting the electron transfer rate at the electrode interface.^29^

**Fig. 6:**
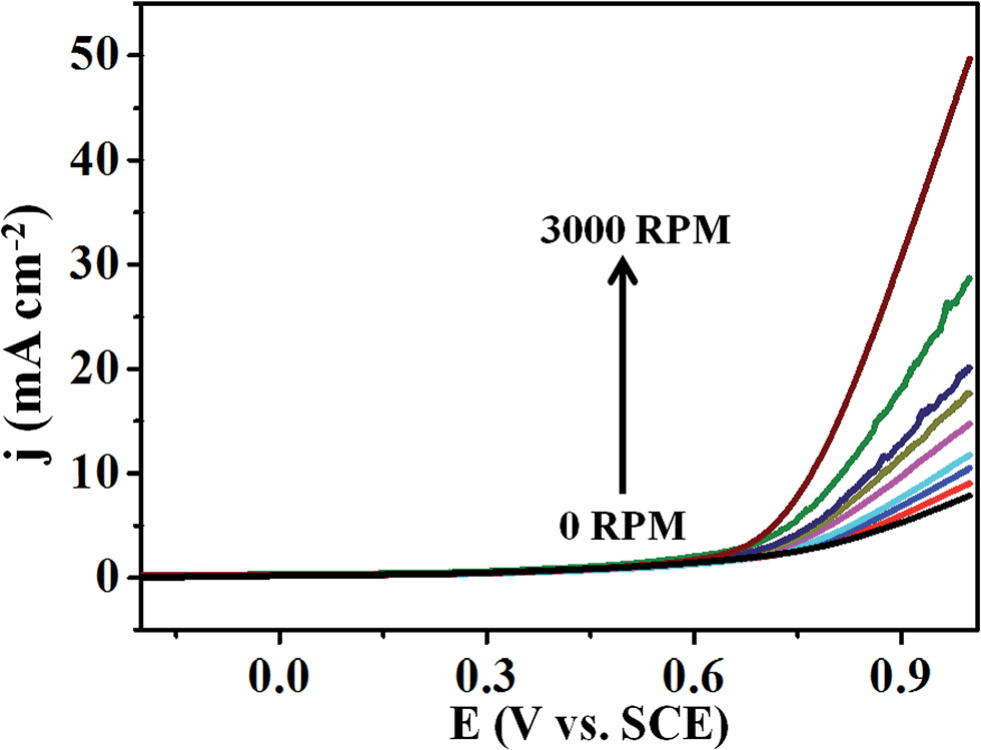
Superimposed LSV curves of T-GO in 0.5 M KOH with different rotating rates from 0 to 3000 rpm.

Moreover, Fig. 6 represents the LSV curves for T-GO at different rotation speeds from 0 to 3000 rpm for OER investigated in 0.5 M KOH. With an increase in rotation speed, the current increases to 0.9 mA cm^−2^ (for 3000 rpm), which indicates diffusion controlled OER on the T-GO based systems.

## 4. Conclusions

In summary, we reported a novel synthetic approach for fabrication of metal free tyramine functionalized GO (T-GO) having a large number of active sites and very high surface area. Efficient electrocatalytic activity towards OER and very good stability was found after tyramine functionalization on GO. XPS, TGA and FTIR data reveal that the existence of tyramine on the surface of GO plays a critical role in tuning the surface structure while increasing the number of active sites on the surface of T-GO. We believe that this enhanced activity after tyramine functionalization, *i.e.* 176 mV overpotential to afford 2 mA cm^−2^ current density and a low Tafel slope of 69 mV dec^−1^, could be due to having tyramine functionalities on the surface, which promotes electron transfer at the interface leading to enhanced OER compared to GO. Hence, T-GO is a promising electrocatalyst having good stability, low cost and effective electrocatalytic performance for OER applications.

## Conflicts of interest

There are no conflicts to declare.

## Supplementary Material

RA-009-C8RA10286D-s001
