# Supplementary material for: Enhanced oxygen evolution reaction on amine functionalized graphene oxide in alkaline medium
Source: RSC Adv. 2019 Feb 22;9(12):6444–51. doi: 10.1039/c8ra10286d (PMC9060968; doi:10.1039/c8ra10286d)
Supplement: RA-009-C8RA10286D-s001 [file RA-009-C8RA10286D-s001.pdf]

Electronic Supplementary Material (ESI) for RSC Advances.
This journal is © The Royal Society of Chemistry 2019

Supporting Information (SI)

Enhanced Oxygen Evaluation Reaction on Amine Functionalized Graphene Oxide in Alkaline Medium

Vijay S. Sapner, Balaji B. Mulik, Renuka V. Digraskar, Shankar S. Narwade and Bhaskar R. Sathe*
Department of Chemistry, Dr. Babasaheb Ambedkar Marathwada University, Aurangabad-431004, Maharashtra, India
Corresponding author E-mail: bhaskarsathe@gmail.com

List of SI:

Figure S1. Scanning electron microscopy image of Graphene-oxide (GO).

Figure S2. XPS survey spectra for C 1s of (a) GO and (b) T-GO respectively.

Figure S2. XPS survey spectra for O 1s of (a) GO and (b) T-GO respectively..

Figure S4. XPS survey spectra for N 1s of T-GO.

Figure S5. TGA superimposed for GO and T-GO.

Figure S6. Cyclic Voltammogram (CV) of GCE, GO and T-GO in 0.5 M KOH at scan 50 mV vs SCE.

Figure S7. TEM images of Graphene Oxide (GO).

Figure S8. AFM topography of Gaphene Oxide (GO).

Scaning electron microscopic study of GO

Morphological study of graphene oxide (GO) as shown in Fig S1 the acitve sufrace sites of GO is less than tyramine-graphne (T-GO) the like wrinkeld folded structure of GO. The size of materials is depends om activity of materials in GO the less active sides becouses the morphology of GO is different from T-GO and electrochemcial activity also different.

High Resolution XPS spectra of GO and T-GO

XPS survey of GO and T-GO materials the XPS spectra of C 1s GO and T-GO are shown in Fig S2 A and B respectively in Fig S2 A the high resolution C 1s spectra of GO materials are show in three individual peaks at

284.8 eV, 286.1 eV and 289.1 eV corresponding to the C-C, C-O and COOH respectivley. In Fig S2 B high resolution C 1s signal was deconvoluted into four individual peaks ascribed to 284.5 eV, 286.1 eV and 288.2 eV corresponding to the C-C, C-O, C-N and C=O respectively. The O 1s excitation resolved into two peaks for both GO and T-GO are shown in Fig S3 A and B. The important peak after the tyramine functinalization of GO the exta XPS peak are observed for the N 1s signal was deconvoluted into two separate peask observed at 399.5 eV, 401 eV corresponding to the (NH-C=O), (C-N) respectively are show in Fig S4.

Cyclic voltammogram (CV) Study of electrocatalyst

Cyclic voltammetric study of Bare GC, GO and T-GO nanomaterials are evalutaed in the alkaline media (0.5M KOH) with a standard three electrode system. Cyclic voltammograms were recorded from -0.2 to 1 V vs SCE and scan rate 50 mV s^{-1} the response of electrocatalyst shown in Fig S6 the T-GO in onset potential observed at ~0.23 V is very lower than the GCE and GO. The electrochemical performance of T-GO is indicated that the electrocatalyst performance of T-GO was excellent for the electrochemical OER applications as compare GO.The current density of electrocatalyst different (i) and different overpotential. The electron transfer reaction is faster in T-GO surface due to the additional funtinalities of sufrace of T-GO than GO. Lower the onset potential to indicate the better the electrocatalyst.

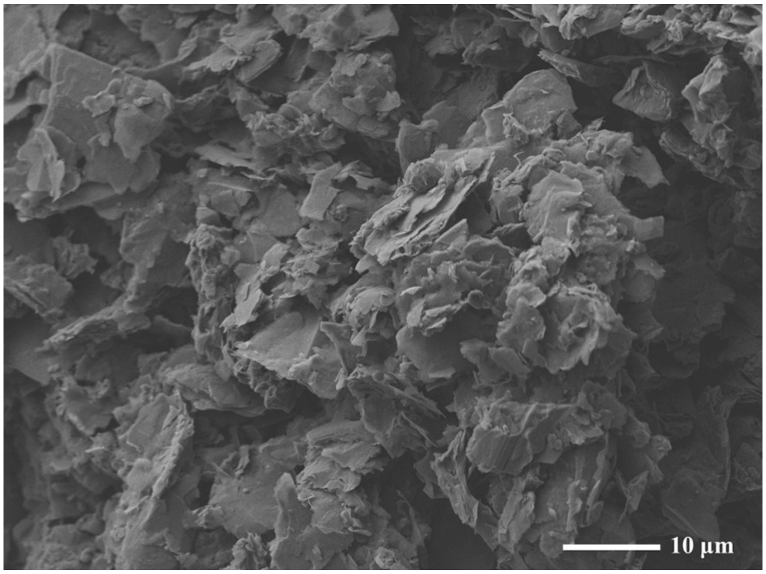

Figure S1. Scanning electron microscopy image of Graphene-oxide (GO).

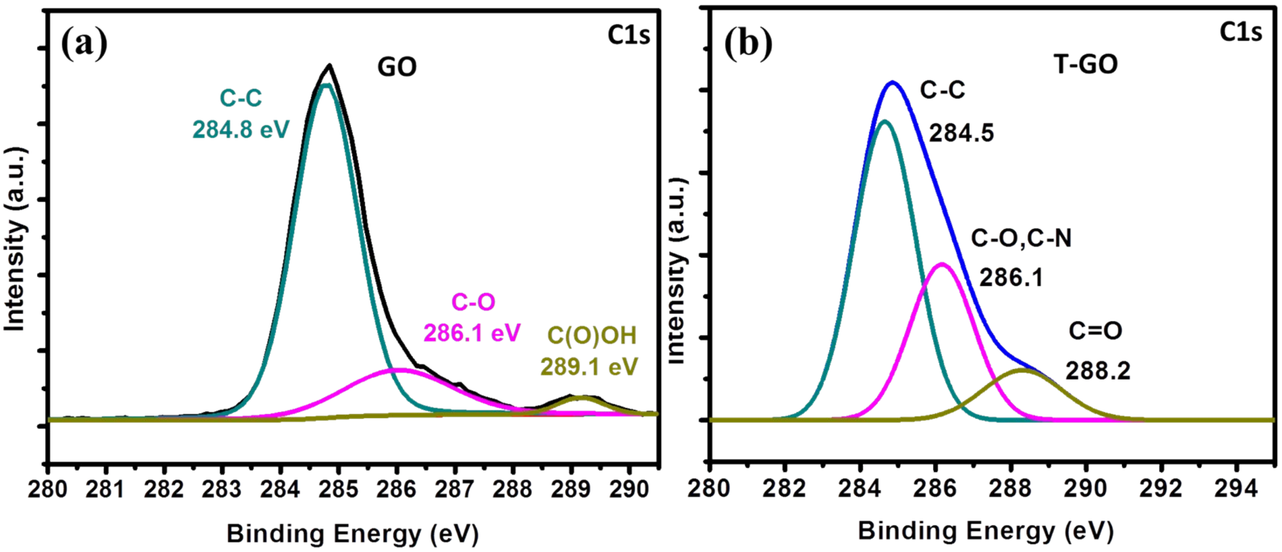

Figure S2. XPS Spectroscopy of all the compound (A) XPS survey spectra of GO C1 s and (B) T-GO C 1s.

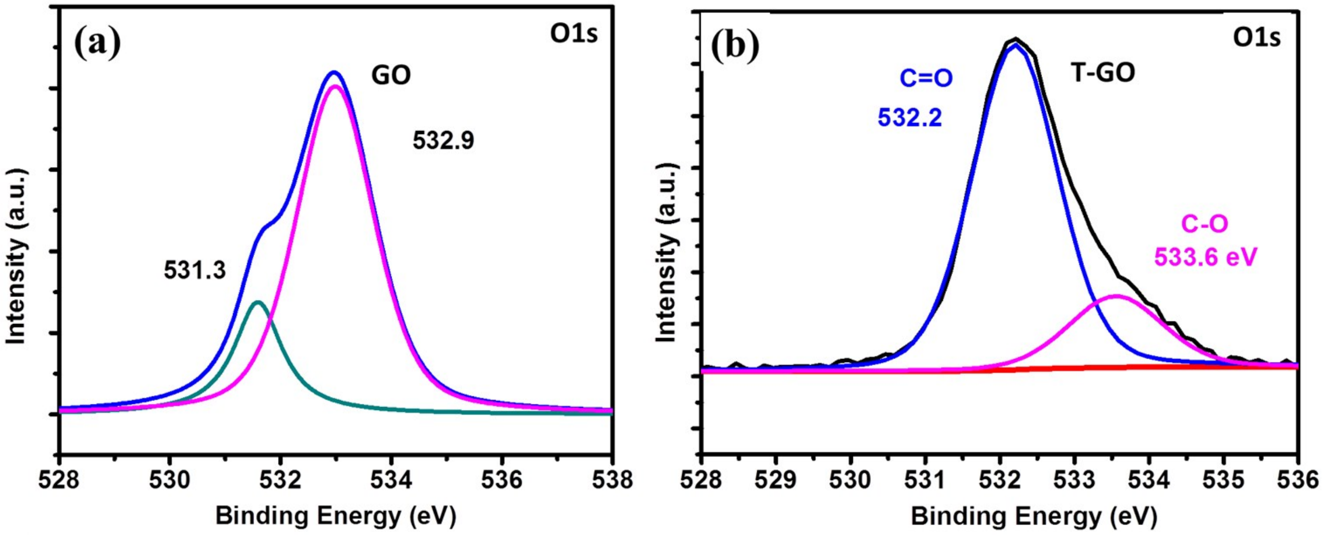

Figure S3. XPS Spectroscopy of all the compound (A) XPS survey spectra of GO O1 s and (B) T- GO O 1s.

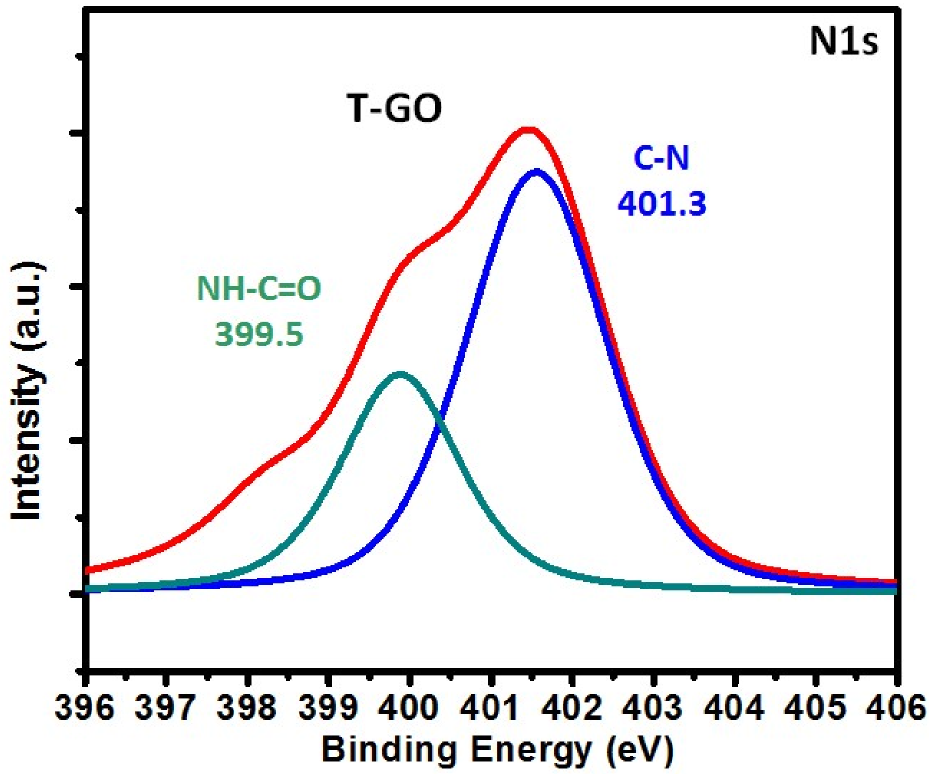

Figure S4. XPS survey spectra of T-GO N 1s.

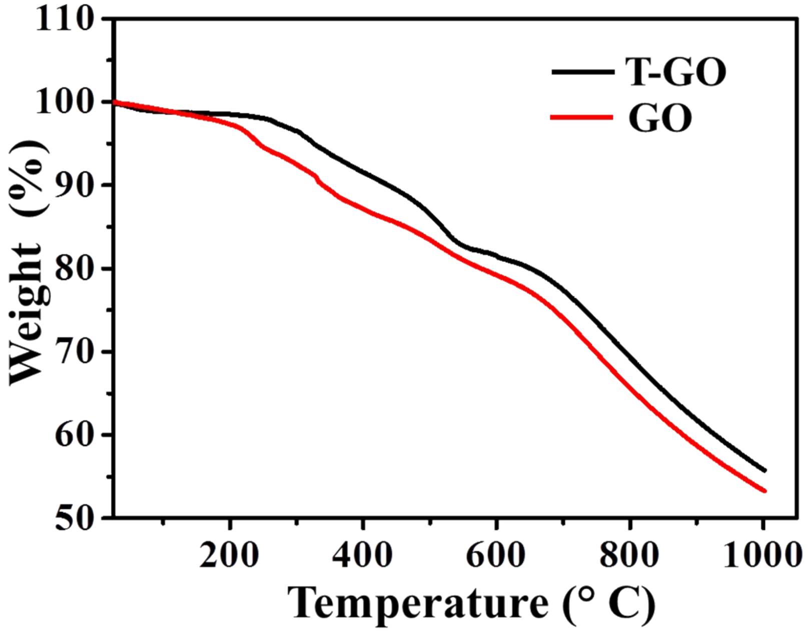

Figure S5. TGA for GO and T-GO

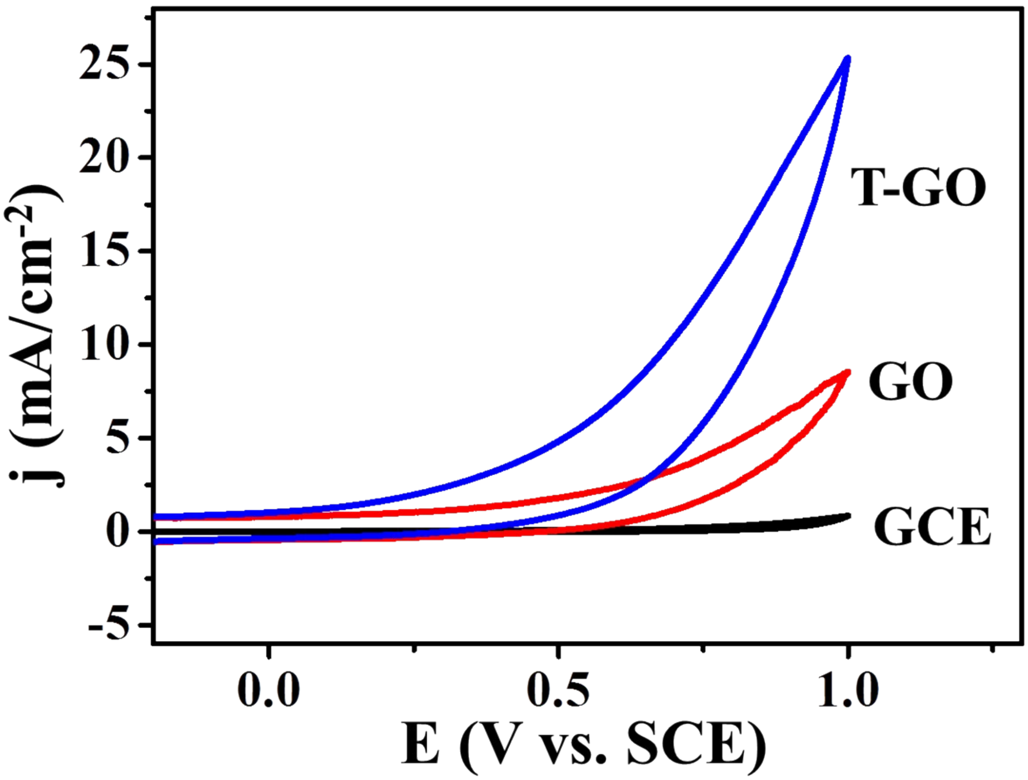

Figure S6. Cyclic Voltammogram (CV) of GCE, GO and T-GO in 0.5 M KOH at scan 50 mV vs SCE

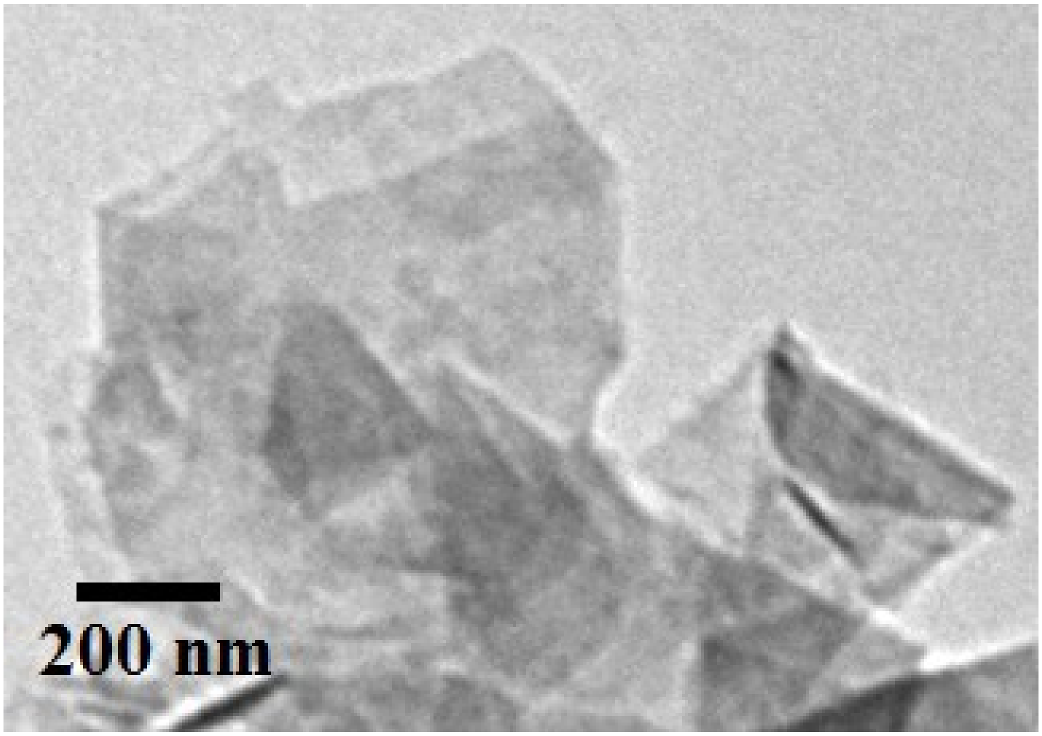

Figure S7. TEM image of Graphene Oxide (GO)

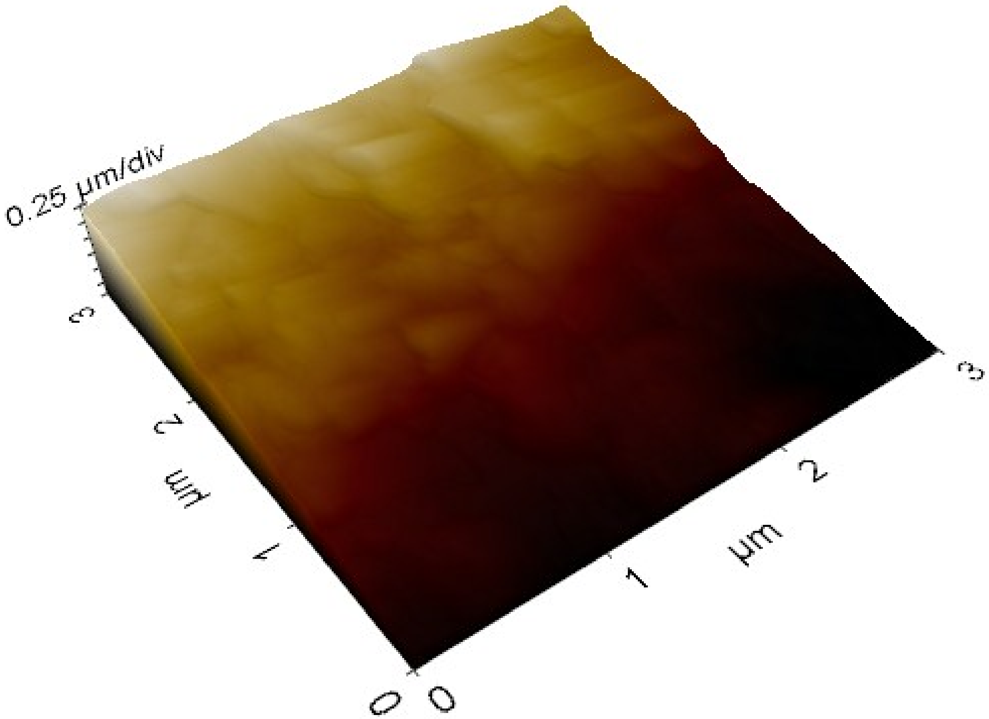

Figure S8. AFM topography of Gaphene Oxide (GO)
